# Maternal anxiety and diet quality among mothers and toddlers from low‐income households

**DOI:** 10.1111/mcn.12992

**Published:** 2020-03-08

**Authors:** Angela C. B. Trude, Maureen M. Black, Pamela J. Surkan, Kristen M. Hurley, Yan Wang

**Affiliations:** ^1^ Growth and Nutrition Division, Department of Pediatrics University of Maryland School of Medicine Baltimore Maryland USA; ^2^ RTI International, Research Triangle Park North Carolina USA; ^3^ Social and Behavioral Intervention Program, International Health The Johns Hopkins Bloomberg School of Public Health Baltimore Maryland USA; ^4^ Center for Human Nutrition, International Health The Johns Hopkins Bloomberg School of Public Health Baltimore Maryland USA

**Keywords:** Healthy Eating Index, maternal anxiety, toddler diet

## Abstract

We evaluated the association between maternal anxiety score and diet quality over time among mothers and toddlers in low‐income families. Longitudinal data were collected from 267 mother–toddler dyads in an obesity prevention trial. Participants were recruited from the Special Supplemental Nutrition Program for Women, Infants and Children and paediatric clinics between 2007 and 2010. Dyads were assessed at study enrolment (Time 1), 6‐month (Time 2), and 12‐month follow‐up (Time 3). On the basis of a 1‐day 24‐hr dietary recall, we estimated maternal and toddler diet quality using the Healthy Eating Index 2015. Anxiety, a time‐varying variable, was assessed via the State–Trait Anxiety Inventory. Associations between maternal anxiety score and maternal and toddler diet quality over time were assessed in adjusted mixed models. Maternal and toddler diet quality were positively correlated (*r* = .48, *p* < .001). Higher maternal anxiety scores were related to lower toddler Healthy Eating Index scores (*b* = −0.51, 95% confidence interval, CI [−0.87, −0.15]) with no significant variation over time. The relation between maternal diet quality and anxiety score varied over time (*b* = 0.28, *p* = .03, for time–anxiety interaction). Higher maternal anxiety scores were associated with lower maternal diet quality at Time 1 (*b* = −0.71, 95% CI [−1.09, 0.34]) and at Time 2 (*b* = −0.51, 95% CI [−0.97, −0.05]), but not at Time 3 (*b* = −0.14, 95% CI [−0.54, 0.26]). Findings suggest that mothers and toddlers exhibited similar low‐quality dietary patterns and that lower diet quality was associated with higher maternal anxiety scores. Approaches to enhance diet quality may consider incorporating anxiety‐reducing strategies into maternal and toddler care and feeding behaviour guidelines.

**Key messages**

- Maternal and toddler diet quality were positively associated.
- Mothers who reported higher anxiety scores had lower diet quality; this association was attenuated over time.
- Higher maternal anxiety scores were associated with lower toddler diet quality; the relationship was consistent over time.
- Toddler diet quality decreased over time, independent of maternal anxiety score.
- Toddlers who had, on average, mothers with higher anxiety scores relative to others tended to have lower diet quality. Future longitudinal studies with repeated measures of anxiety and diet should consider assessing the associations on a shorter timescale (i.e., daily, weekly, or monthly) to inform temporal specificity of anxiety–dietary intake relations.

## 1 INTRODUCTION

In the United States, prevalence of high weight for length (>97th percentile) among infants and toddlers (<24 months old) reached 7.1% in 2011–2012 (Ogden, Carroll, Kit, & Flegal, 2014), two times as high as other developed countries such as Canada (3.5%) (Furlong et al., 2016). Excess weight gain is a public health concern because children who are overweight and obese are at increased risk of becoming obese and of developing noncommunicable diseases as adults, particularly if their parents are overweight or obese (Llewellyn, Simmonds, Owen, & Woolacott, 2016; Simmonds, Llewellyn, Owen, & Woolacott, 2016). Paediatric obesity often begins with rapid weight gain early in life (Zheng et al., 2018), and parents, specifically mothers, have an important influence on children's weight‐related behaviours (Cameron et al., 2011).

Maternal psychological well‐being has been understudied among maternal risk factors for child feeding practices, including food intake and diet quality (de Jong, Visscher, HiraSing, Seidell, & Renders, 2015; Gillman et al., 2008; Woo Baidal et al., 2016). Most studies investigating the association between maternal psychosocial well‐being and diet‐related behaviours among infants and young children have focused on maternal depressive symptoms (Barthel et al., 2017; Benton, Skouteris, & Hayden, 2015; Morrissey & Dagher, 2014; Savage & Birch, 2017). Longitudinal relations have been described between maternal depression reported 1 year post‐partum and higher child body mass index (BMI; Benton et al., 2015), low physical activity levels (Duarte, Shen, Wu, & Must, 2012), and age‐inappropriate child feeding practices (Thompson & Bentley, 2013). Although maternal anxiety and depression often co‐occur, anxiety may have unique patterns and relations with weight gain that warrants further investigation (Nawa, Black, Araya, Richiardi, & Surkan, 2019).

The toddler stage (12–36 months) can be challenging for mothers, associated with toddlers' rapidly changing mood shifts, picky eating, food refusal, and food neophobia (Cole, An, Lee, & Donovan, 2017). Emotional distress has been documented among mothers of toddlers who are frequently concerned about the quantity of foods consumed and in turn provide food of poor nutritional quality (Brown et al., 2016; Harris, Ria‐Searle, Jansen, & Thorpe, 2018).

Mothers from socio‐economically disadvantaged households may be particularly vulnerable to lower psychological well‐being (i.e., anxiety, depression, and stress) due to additional concerns about food waste, time, and financial constraints (Goodell, Johnson, Antono, Power, & Hughes, 2017). Individuals in low‐income households are also at greater risk for low diet quality and obesity (Ogden et al., 2017). Despite some recent findings showing a temporal improvement in diet quality on the Healthy Eating Index (HEI) among U.S. adults (Wilson, Reedy, & Krebs‐Smith, 2016), the gap in dietary quality between high‐ and low‐income individuals has widened in the past decade (D. D. Wang et al., 2014). Examining understudied risk factors, such as anxiety, and the relation to diet quality among families of low‐income backgrounds may increase our understanding of socio‐economic health disparities.

Anxiety in mothers of toddlers has the potential to influence parenting behaviours. Anxiety has been associated with maternal overstimulation due to lower sensitivity to cues that their behaviours are intrusive (Kaitz & Maytal, 2005) or understimulation due to general feelings of worry and apprehension (withdrawn behaviour) associated with low parenting self‐efficacy (Seymour, Giallo, Cooklin, & Dunning, 2015). Maternal anxiety has been associated with forceful feeding practices at 12 months of age (Farrow & Blissett, 2005; Hurley, Black, Papas, & Caulfield, 2008). However, to date, studies of maternal anxiety and toddler diet have been primarily cross‐sectional and results have been mixed, showing no association with diet quality among 7–12 months old infants (Hurley, Black, Merry, & Caulfield, 2015) but a positive correlation with unhealthful dietary patterns among 18‐month‐old children (Ystrom, Barker, & Vollrath, 2012).

Maternal anxiety may influence mothers' own weight‐related behaviours (Emerson, Hurley, Caulfield, & Black, 2017), which in turn play a negative influence on her child's behaviours through role modelling and poor mother–toddler interactions (Reck, Tietz, Müller, Seibold, & Tronick, 2018). Diet quality may also be associated with mental health, as diets low in essential minerals have been associated with anxiety related to inflammation, oxidative stress, and neuroplastic changes (Młyniec, Gaweł, Doboszewska, Starowicz, & Nowak, 2017). In both directions, maternal and toddler diets could be disrupted. Given that anxiety is a transient state for some mothers, but persistent for others (i.e., a trait; Barthel et al., 2016), limited information can be gained from cross‐sectional studies. During toddlerhood, children are forming their food preferences and exercising their independence, thus dynamically shaping parent–child mealtime interactions and parental feeding concerns. Hence, longitudinal studies can enhance our understanding of the association between maternal anxiety and diet‐related behaviours, examine the stability of associations over time, and inform practice (Field, 2018).

Temporal data provide multiple data points for each individual, enabling the investigation of how changes in an individual mother's state of anxiety relate to diet quality (time‐varying, occasion‐dependent relation) at different developmental stages of toddlerhood. This time‐varying relation is particularly important to study because anxiety may be time‐varying or constant (Howard, 2015). In addition, a stable relation over time would enable between‐person associations (the population‐averaged estimate of the relation between overall maternal anxiety and overall diet) to be disaggregated from within‐person associations (the person‐specific relation between the fluctuation of maternal anxiety from her own average and the mother/toddler's diet) (Dunton, 2018). Thus, identifying whether instability or consistency in maternal anxiety predicts toddlers' diet quality can better inform behaviour interventions.

This study had the following objectives:

1. to examine the association between maternal state anxiety score and maternal diet quality over time;
2. to examine the association between maternal state anxiety score and toddlers' diet quality over time; and
3. to disaggregate the within‐person and between‐person associations between maternal state anxiety score and diet quality, if relation does not differ over time.

## 2 SUBJECTS AND METHODS

### 2.1 Study design

Longitudinal data were obtained from a randomized obesity prevention trial of 277 low‐income mother–toddler dyads assigned to (a) safety (attention control), (b) maternal lifestyles (diet/physical activity), or (c) responsive parenting. Study subjects were recruited from a Women, Infants, and Children (WIC) clinic located in a semiurban community and a primary care paediatric clinic serving a low‐income urban community of the Mid‐Atlantic region of the United States between 2007 and 2010 (Black et al., Under review; Wang, Gielen, Magder, Hager, & Black, 2018). The intervention was based on social cognitive theory (Bandura, 2004) and delivered by trained interventionists in community sites (four group sessions and a final review/celebration group session) and over the phone (three individual sessions). Assessments occurred at study enrolment (Time 1), 6‐ (Time 2 [T2]), and 12‐months after enrolment (Time 3 [T3]). Each evaluation consisted of two parts: an in‐person laboratory visit to assess weight, height, and self‐reported measures and a home visit (a week later) to conduct the 24‐hr diet recall. Further details can be found elsewhere (Y. Wang et al., 2018).

### 2.2 Subjects

Eligibility criteria for mothers included being the biological mother of an eligible toddler, being aged >18 years, being not pregnant, and having no plans of leaving the area in the coming year. Eligibility criteria for toddlers included being aged 12–32 months, being born at term, and had a birthweight of >2,500 g.

### 2.3 Data collection

Trained research assistants assessed maternal and toddler anthropometry, diet, and maternal anxiety scores at the three time points. Written informed consent was obtained from each mother prior to Time 1 data collection. This study was reviewed and approved by the ethical review boards of a university and a state department of health.

**Figure 1:**
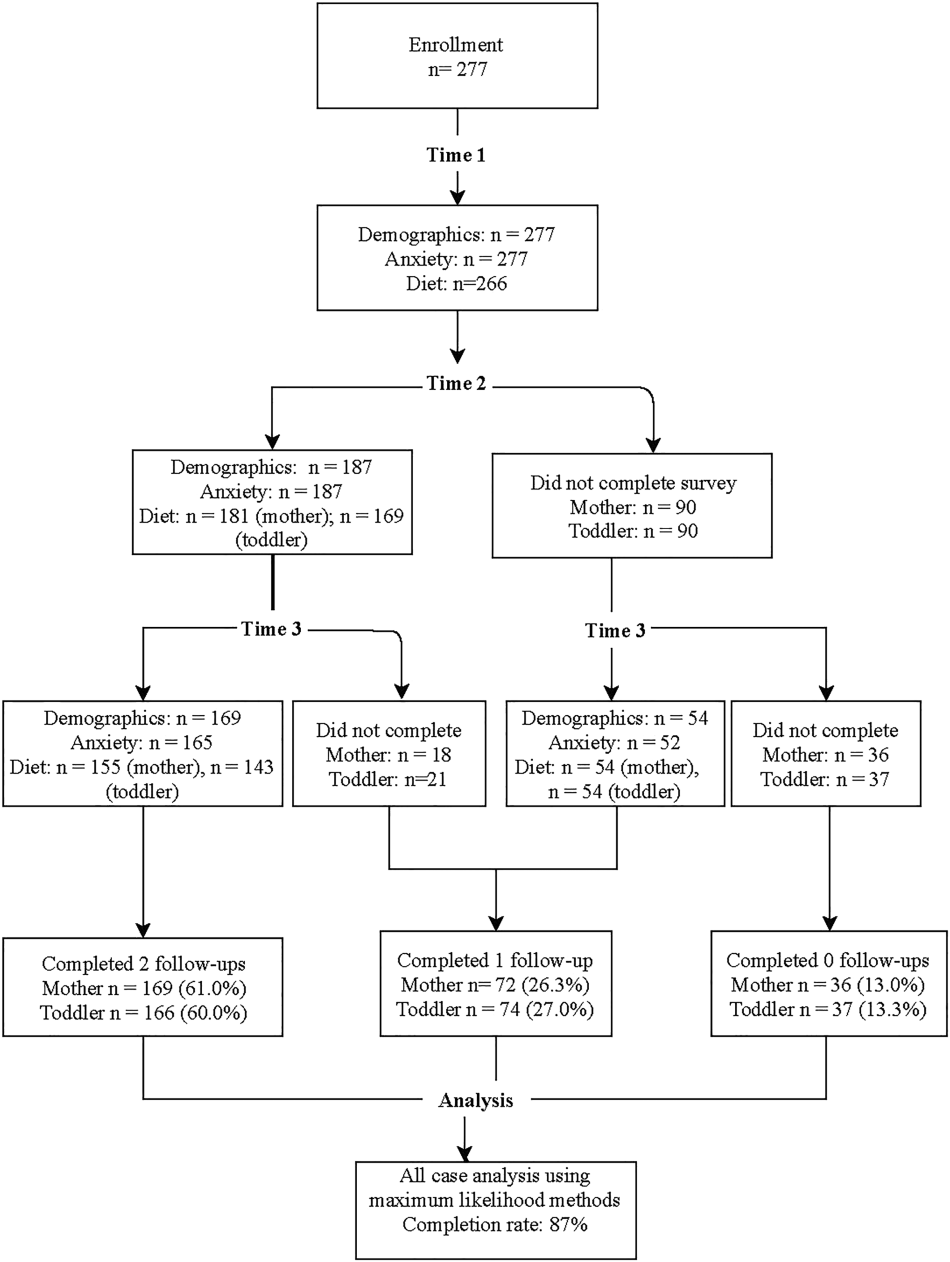
Flow diagram of study participants in the longitudinal assessment

A total of 277 mother–toddler dyads were assessed at Time 1; 266 had complete dietary recalls. At Time 2 (6 months after enrolment), 187 mother–toddler dyads were interviewed; 181 and 171 mothers and toddlers had completed dietary recalls, respectively. At Time 3, we interviewed 224 mother–toddler dyads; 24‐hr dietary recalls were complete for 213 mothers and 202 toddlers. Figure 1 illustrates the inclusion of study participants into the analytical sample. No patterns of dropout were observed by baseline demographic characteristics or treatment group; thus, data were considered to be missing completely at random.

### 2.4 Measures

#### 2.4.1 Maternal and toddler diet quality

We used an interviewer‐administered computerized multiple‐pass method (USDA AMPM) to collect data on intake of foods and beverages consumed within a 24‐hr period at three time points in mothers and toddlers (maternal reported). The HEI‐2015 was estimated on the basis of the 24‐hr diet recall, assessing the adherence to the 2015–2020 Dietary Guidelines for Americans (DGA) with high validity and reliability (Kirkpatrick et al., 2018; Krebs‐Smith et al., 2018; Reedy et al., 2018).

The HEI scoring algorithm method adjusts for total energy consumption to derive 13 HEI components and HEI total score for both mother and toddler. Nine food components assessed adequacy of intake (namely, total fruit, whole fruit, total vegetables, greens and beans, whole grains, dairy, total protein foods, seafood and plant proteins, and fatty acids), and higher scores indicate higher consumption. In other four food components, lower intakes are desirable (refined grains, sodium, saturated fats, and added sugars) and are coded such that higher scores indicate lower consumption. The 13 components were summed to generate the HEI total score for mothers and toddlers (0–100 points), where higher scores denote better diet quality. HEI components, range of possible scores, and average points over time are shown in Table S1.

#### 2.4.2 Maternal anxiety symptoms score

Maternal anxiety symptoms were measured using the six‐item short form of the state scale for the Spielberger State–Trait Anxiety Inventory (STAI) (Spielberger, 1989) at three time points. The state anxiety scale includes three anxiety‐present (i.e., “I am tense,” “I feel upset,” and “I am worried”) and three anxiety‐absent items (i.e., “I feel calm,” “I am relaxed,” and “I am content”). This questionnaire has demonstrated acceptable reliability (Cronbach's alpha = .82) and concurrent validity compared to the full version (Marteau & Bekker, 1992). A score of 12 points or higher on the short‐form STAI is used to indicate anxiety in the screening (Spielberger, 1989). All items were rated on a 4‐point scale from *not at all* to *very much so.* Anxiety‐absent items were reverse coded, and the sum of the total score was calculated (possible range 6 to 24 points, where higher scores represent more severe anxiety). Anxiety score in this study was treated in its continuous form.

#### 2.4.3 Anthropometry

Both mothers and toddlers had their heights (or lengths) measured in centimetres using a Shorr measuring board (Shorr Productions, Olney, MD) and weight (kg) using a Tanita 1584 Baby Scale (Tanita, Tokyo, Japan) for toddlers and a Tanita 300GS for mothers measured in triplicate. All measures were assessed at three time points by trained research staff. BMI was calculated by dividing weight (kg) per height squared (m^2^) for mothers. For toddlers, we used age‐ and sex‐specific World Health Organization growth charts to calculate BMI *z*‐scores (Grummer‐Strawn, Reinold, & Krebs, 2010). BMI *z*‐scores were used instead of weight‐for‐length *z*‐scores due to the varying age of the children (range 12–60 months) attributed to the longitudinal design of the study following the American Academy of Pediatrics recommendations (Daniels & Hassink, 2015). A high correlation between weight‐for‐length and BMI‐*z* has been reported among children younger than 2 years, indicating that the use of BMI is appropriate for both early infancy and toddlerhood (Furlong et al., 2016; Roy et al., 2016).

#### 2.4.4 Sociodemographic

Sociodemographic characteristics of toddlers and their mothers were collected at Time 1, Time 2, and Time 3 assessments (see Table 1). Age was calculated using the date of birth and the date of the interview (months for toddlers; years for mothers) and treated as a continuous variable. Mothers reported their own and their toddler's race/ethnicity as Non‐Hispanic White, Non‐Hispanic Black, Hispanic/Latino, and other. Mothers reported their highest education level, and it was categorized into having completed or not having completed high school. At Time 1, mothers also reported their toddler's birth measures such as gestational age, and weight and length at birth.

**Table 1:** Descriptive information on maternal and child characteristics, weight status, diet quality, and maternal mental health symptoms

Characteristic	Time 1	Time 2	Time 3
	Mean (SD) (%)	Mean (SD) (%)	Mean (SD)(%)
Maternal	(*n* = 277)	(*n* = 187)	(*n* = 223)
Age (years)	27.3 (6.2)	28.1 (6.5)	28.9 (6.5)
Marital status (married)	28.2		
Completed high school	80.9		
Poverty ratio (<1.0)^a^	68.5		
Average number of children in the household	2.4 (1.2)		
Has another child younger than toddler in the study	18.4		
Household food insecurity^b^	29.4	30.3	24.5
WIC participation	87.0	81.1	69.3
Body mass index (kg m^−2^)	31.8 (9.5)	31.7 (9.7)	32.6 (9.5)
Race/ethnicity			
Black	67.5		
White	26.7		
Hispanic	2.9		
Other	2.9		
Mental health symptoms			
Anxiety score^c^^,^ e	10.2 (3.7)	10.0 (3.8)	10.4 (3.9)
HEI‐2015^e^	47.9 (12.1)	48.3 (12.1)	47.5 (13.2)

Child	(*n* = 277)	(*n* = 187)	(*n* = 219)
Age (months)	20.1 (5.5)	26.7 (5.7)	34.6 (8.8)
Sex (female)	46.9		
First child	48.1		
Twin or triplet	6.8		
Body mass index (*z*‐score)^d^	0.5 (1.1)	0.6 (1.0)	0.6 (1.1)
HEI‐2015^e^	54.1 (10.5)	53.4 (11.5)	52.6 (11.8)
Ever breastfed	51.5		
Birth measures (maternal reported)			
Premature birth	9.8		
Birthweight (grams)	218.3 (119.0) (4.2)		
Birth length (cm.)	49.8(7.1)		

Abbreviations: HEI, Healthy Eating Index; SD, standard deviation; Time 1, enrolment; Time 2, 6‐month follow‐up; Time 3, 12‐month follow‐up; WIC, Maryland Special Supplemental Nutrition Program for Women, Infants and Children.

a The income‐to‐poverty ratio was calculated according to the U.S. Census Bureau, Weighted Average Poverty Thresholds, 2010.

b Assessed with six‐item U.S. Food Security Scale. Food insecurity is defined if score is between 2 and 6 points. Change over time analysis used total food security score (0–6) as a continuous variable.

c Anxiety was assessed with the six‐item Spielberger State–Trait Anxiety Inventory (possible range 6–24 points).

d Body mass index was calculated for age‐ and sex‐specific *z*‐score according to the World Health Organization Child Growth Standard.

e Multilevel growth models assessed change over time: maternal anxiety (*b* for Time 2 vs. Time 1 = −0.04, 95% CI [−0.57, 0.49]; *b* for Time 3 vs. Time 1 = 0.21, 95% CI [−0.29, 0.71]), maternal HEI (*b* for Time 2 vs. Time 1 = −0.11, 95% CI [−1.90, 2.12]; *b* for Time 3 vs. Time 1 = −0.55, 95% CI [−2.45, 1.35]), and toddler HEI (*b* for Time 2 vs. Time 1 = −0.71, 95% CI [−2.73, 1.30]; *b* for Time 3 vs. Time 1 = −1.48, 95% CI [−3.39, 0.42]).

Household food security was assessed with the six‐item U.S. Food Security Scale, considered an appropriate substitute of the 18‐item module with high sensitivity and specificity for identifying food‐insecure households (Blumberg, Bialostosky, Hamilton, & Briefel, 1999). Household food security status was coded using recommended cut‐points: 2 to 6 affirmative answers for food insecurity and 0 to 1 for household food security. WIC enrolment status was reported by mothers at the three time points.

The income‐to‐poverty ratio was calculated using the weighted average poverty thresholds by the U.S. Census Bureau (2010). A poverty ratio below 1 indicates that the average income for each respective family size is below the federal poverty threshold. The categorized income‐to‐poverty ratio variable was included as a covariate in all analyses.

### 2.5 Data analysis

All variables were processed in QDS™ Warehouse Manager (NOVA Research Company, MD, USA). All statistical analyses were conducted using Stata SE 15.1 for Windows software (StataCorp, College Station, TX, USA). For all analyses, the 95% confidence intervals (CIs) were reported. Statistical significance was defined by a *p* value of <.05.

Sociodemographic, anthropometric, dietary, and maternal anxiety variables were examined to describe the study population. Univariate distributions were examined to identify extreme values (outliers) and skewed continuous distributions; none were identified. Means, standard deviations (SDs), and prevalence (%) were estimated for key baseline descriptors. Multilevel growth models were used to assess change in mother and toddler's diet, and maternal anxiety score over time.

Multilevel mixed‐effect models were performed to assess the association between maternal anxiety score in relation to maternal and toddler diet quality using maximum likelihood estimation, with random intercepts to account for the clustering of the repeated measures over time within the same individual. Our model specification checks, including assessment of model residuals, revealed that all normality assumptions were met when treating the outcomes in their continuous form. The intraclass correlation coefficients between repeated measures within each individual were .31 for mothers and .14 for toddlers, without including any predictor in the mixed‐effect models.

First, we assessed the association between anxiety (time‐varying predictor) and diet quality over time. To assess whether the association varied over time, we included an interaction term between time of assessment and maternal anxiety score. On the basis of this model, we estimated and plotted the predicted mean diet quality over time by each level of maternal anxiety score. Because there is variation in the toddler's age at each time point, we conducted sensitivity analyses that assessed children's age (continuous) instead of the time point in the interaction.

Then, if the association between diet and maternal anxiety score did not systematically change as a function of time, we disaggregated the within‐person and between‐person effects of maternal anxiety score and diet quality (Curran & Bauer, 2011), using the method of person‐mean centring, as recommended (Howard, 2015).

For the most parsimonious model, we selected the following covariates: maternal age (continuous), toddler age (continuous) and sex (male = 0, female = 1), maternal BMI (continuous) and toddler BMI (*z*‐score), and poverty rate (as time‐varying variables) and intervention group (time invariant). Variables were chosen on the basis of associations reported previously and significant associations with anxiety and diet quality. Sensitivity analyses were conducted additionally controlling for parity and age of the youngest child. For all the analyses, no significant effect by intervention group on diet quality was found in a three‐way interaction term (e.g., for maternal diet quality, a *p* value of Anxiety * Time 2 * Group 2 = .65; Anxiety * Time 2 * Group 3 = .54; Anxiety * Time 3 * Group 2 = .95; and Anxiety * Time 3 * Group 3 = .59); thus, we combined the intervention and control groups, and the intervention group was treated as a covariate in the models.

## 3 RESULTS

### 3.1 Sample characteristics

On average, toddlers were 20.1 months (SD ±5.5), and mothers were 27.3 years old (SD ±6.2) at baseline. The majority of families lived below the poverty threshold (68.5%) and were enrolled in the WIC programme throughout the study. Mothers had an average of 2.4 children in the household, and living below the poverty line was positively associated with having more children in the household, compared with living above the poverty line (mean 2.6 [SD 0.9] vs. 2.0 [0.1], *t* test *p* value <.001). Mothers and toddlers exhibited similar dietary patterns (*r* = .48, *p* < .001) of low diet quality (maternal HEI at Time 1 = 47.9 points; toddler HEI at Time 1 = 54.1 points). Maternal anxiety scores were on average 10 points on the short‐STAI (SD 3.7; observed range 5–21), meaning that most mothers reported *not at all* or *somewhat* anxious to each item. One third of mothers presented elevated anxiety scores (>12 points) at baseline. About half of the sample were first‐time mothers (48.1%), and anxiety scores did not differ between first‐time and other mothers (*p* = .77). There was no statistically significant change in either maternal/toddler diet or maternal anxiety score over time of assessment.

### 3.2 Associations between maternal anxiety score and maternal diet quality over time

Maternal diet quality varied by maternal anxiety score over time (*b* = 0.28, *p* = .03, for the interaction). Higher maternal anxiety scores were associated with lower maternal diet quality at Time 1 (*b* = −0.71, 95% CI [−1.09, −0.34]) and at Time 2 (*b* = −0.51, 95% CI [−0.97, −0.05]), but not at Time 3 (*b* = −0.14, 95% CI [−0.54, 0.26]; Table 2). Our results remained the same when additionally controlling for parity and age of the youngest sibling in the model (Table S2).

**Table 2:** Associations between maternal anxiety score and diet quality of mothers and toddlers across three time points

Model	Maternal anxiety score							
	Anxiety		Difference in beta between Time 1 and Time 2		Difference in beta between Time 1 and Time 3		Difference in beta between Time 2 and Time 3	
	*b*	95% CI	*b*	95% CI	*b*	95% CI	*b*	95% CI
Maternal HEI‐2015 total score^a^								
Time 1	−0.71	[−1.09, −0.34]	Reference		Reference			
Time 2	−0.51	[−0.97, −0.05]	0.20	[−0.35, 0.76]			Reference	
Time 3	−0.14	[−0.55, 0.26]			0.57	[0.05, 1.10]	0.37	[−0.21, 0.95]
Toddler HEI‐2015 total score^a^								
Time 1	−0.51	[−0.87, −0.15]	Reference		Reference			
Time 2	0.01	[−0.44, 0.45]	0.52	[−0.04, 1.09]			Reference	
Time 3	−0.18	[−0.57, 0.21]			0.33	[−0.19, 0.86]	−0.20	[−0.78, 0.40]

Abbreviations: *b*, the average marginal effect of change in HEI‐2015 total score that is produced by a one‐unit increase in maternal anxiety at a given time point; CI, confidence interval; HEI‐2015, Healthy Eating Index 2015; Time 1, enrollment; Time 2, 6‐month follow‐up; Time 3, 12‐month follow‐up.

a Hierarchical models controlled for maternal and toddler's age and sex, maternal body mass index (kg m^−2^), toddler body mass index *z*‐score, living at or below the poverty line, and intervention group.

### 3.3 Associations between maternal anxiety score and toddler diet quality over time

The relation between maternal anxiety score and toddler diet did not vary over time (*b* for anxiety‐time interaction = 0.17, 95% CI [−0.09, 0.44]). Therefore, we restricted the relation to be equal over time. Higher maternal anxiety score was associated with lower toddler diet quality (*b* = −0.27, 95% CI [−0.51, −0.03]), meaning that for each additional 6‐point increase in maternal anxiety score (shift from one response category in the Likert scale to the next for all the six STAI items), there was a decrease in 1.6 HEI points for toddlers.

### 3.4 The within‐ and between‐persons effect of maternal anxiety score on toddler diet quality

Because the association between maternal anxiety score and toddler diet did not vary over time, we disaggregated the between‐person relation and within‐person relation. The between‐person effect of maternal anxiety score was statistically significant, indicating that mothers who were on average more anxious over time had 0.4 points lower HEI scores for their toddlers (*b* = −0.37; 95% CI [−0.65, −0.10]; Table 3). The within‐person effect was not statistically significant, suggesting that for a specific mother–toddler dyad, the mother experiencing higher than usual anxiety did not report lower than usual HEI scores for her toddler on that occasion.

**Table 3:** Within‐ and between‐person effects of maternal anxiety score on toddler diet quality

Fixed effects	Maternal anxiety symptoms^a^	
	*b*	95% CI
Intercept	56.68	[51.01, 62.37]
Within persons	−0.05	[−0.46, 0.35]
Between persons	−0.37	[−0.65, −0.10]

*Note.* Model: Toddler HEI‐2015 total score.

Abbreviations: *b*, estimate; CI, confidence interval; HEI‐2015, Healthy Eating Index 2015.

a Hierarchical models controlled for maternal and toddler's age and sex, maternal body mass index (kg m^−2^), toddler body mass index *z*‐score, poverty, and intervention group.

### 3.5 Sensitivity analysis

**Figure 2:**
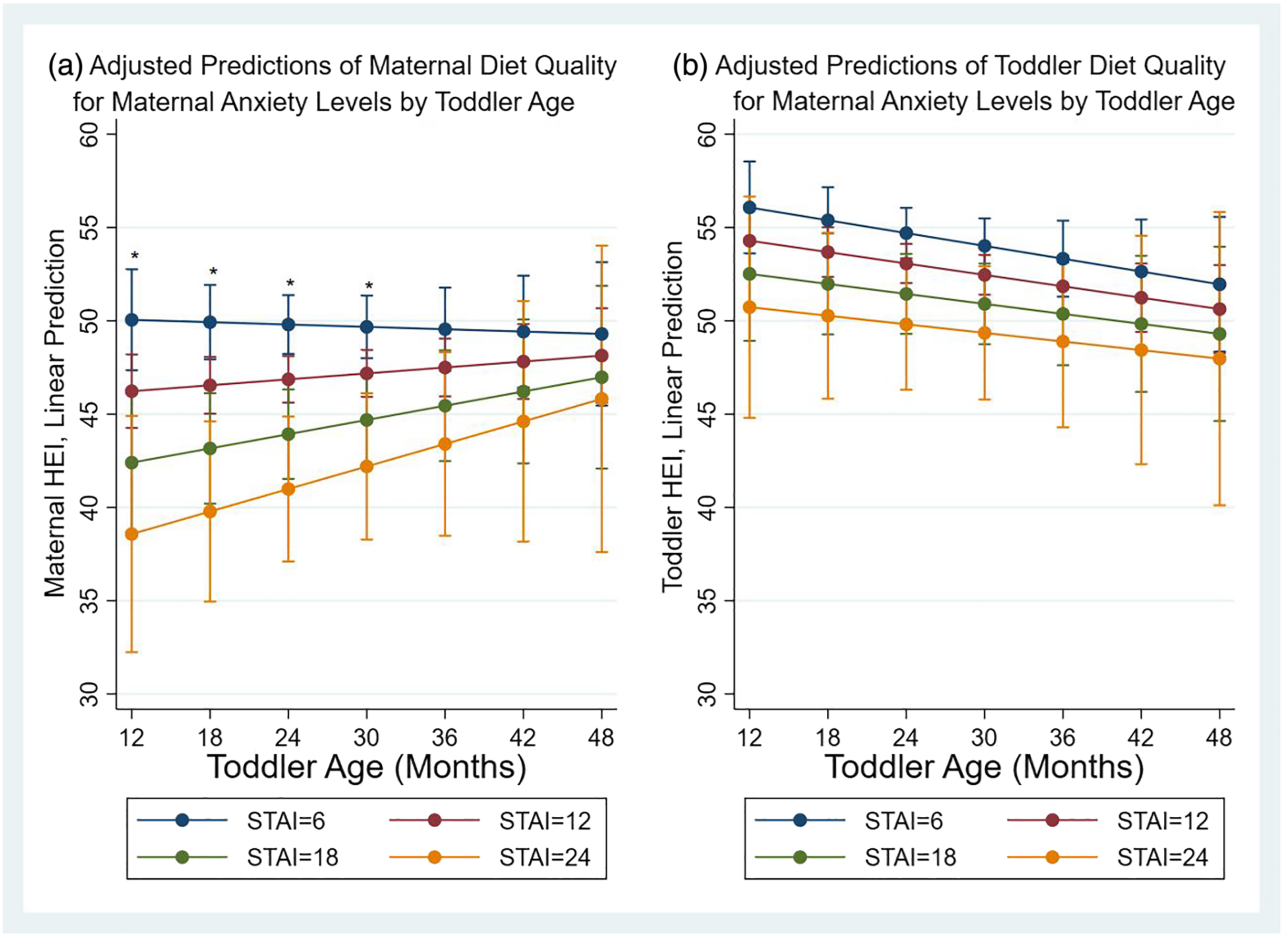
Adjusted predictions of maternal diet quality (Healthy Eating Index [HEI]‐2015) and toddler diet quality (HEI‐2015) for the interaction between maternal anxiety levels over time (toddler age). Hierarchical models controlled for maternal and toddler's age and sex, maternal body mass index (kg m^‑2^), toddler BMI *z*‐score, poverty, and intervention group. Anxiety was assessed with the six‐item Spielberger State–Trait Anxiety Inventory (STAI). Predictions for STAI points of 6, 12, 18, and 24 were chosen to illustrate anxiety symptoms for mothers who answered on average *not at all*, *somewhat*, *moderately so*, and *very much so*, respectively, for anxiety‐present questions. (a) Asterisks represent that maternal diet quality is statistically significantly higher among mothers with less anxiety symptoms (STAI = 6) compared with those with multiple anxiety symptoms (STAI = 24): at 12 months (STAI 6: B = 50.1, 95% CI [47.3, 52.7]; STAI 24: B = 38.6, 95% CI [32.2, 44.9]); at 18 months (STAI 6: B = 49.9, 95% CI [47.9, 51.9]; STAI 24: B = 39.7, 95% CI [34.9, 44.6]); at 24 months (STAI 6: B = 49.8, 95% CI [48.2, 51.4]; STAI 24: B = 40.9, 95% CI [37.1, 44.8]); and at 30 months (STAI 6: B = 49.6, 95% CI [47.9, 51.3]; STAI 24: B = 42.1, 95% CI [38.2, 46.1]). (b) Overall, toddler diet quality decreased by 0.1 HEI points per unit increase in toddler's age per month, *b* = −0.11 (0.05), 95% CI [−0.20, −0.02]. The interaction term between maternal anxiety (continuous) and toddler age (months) was not statistically significant, *b* = 0.01 (0.01), 95% CI [−0.02, 0.02]

Our sensitivity analysis showed that maternal diet quality was lower among mothers who had higher anxiety scores and younger toddlers (Figure 2a), and this relation was statistically significantly different when toddlers were between ages 12 and 30 months (approximately Time 1 and Time 2 assessments).

Figure 2b depicts the sensitivity analysis using toddler's age as the time metric in the interaction term. Overall, toddler diet quality decreased by 0.1 HEI points per unit increase in toddler's age in months, *b* = −0.11 (±0.05), 95% CI [−0.20, −0.01], and was lower among toddlers who had mothers with higher anxiety scores compared with those with lower anxiety scores, although this difference did not vary statistically over time.

## 4 DISCUSSION

This study adds to our understanding of the relation between maternal anxiety and maternal and toddler's diet quality over time in a sample of low‐income WIC‐eligible families. Our findings suggest that maternal diet quality was lower among mothers who reported higher anxiety scores when their toddlers were between 12 and 30 months old, but differences were attenuated when children reached age 36 months.

We found that toddlers' diets were of low quality, with slightly over 50 HEI points. Even though HEI scores are usually not calculated for children younger than 2 years (as this age group is not currently included in the DGA), similar studies have calculated the HEI score for this population (Au et al., 2018). A nationally representative study reported diet quality at 64 HEI points among WIC toddlers between 13 and 24 months (Au et al., 2018). In our sample of low‐income toddlers, HEI decreased for each additional month of age, as they transition from complementary feeding to family meals. This finding was not corroborated by a recent review that reported a positive association between family meals and child diet quality (Verhage, Gillebaart, van der Veek, & Vereijken, 2018). It is possible that children's diets resemble their mother's diets as they transition to family foods. Furthermore, toddlers' HEI scores of sodium, added sugar, and refined grains were consistently low across the three assessments (Table S1), which might be driving their overall HEI score down.

In our study, higher maternal anxiety score using the STAI screener was associated with lower maternal and toddler diet quality. Although maternal anxiety has been associated with negative parenting (Seymour et al., 2015), restrained eating attitudes (Emerson et al., 2017), and adverse health outcomes (Singh, 2014), our study is novel in reporting on associations between anxiety and maternal diet among a unique population of mothers of toddlers. Low‐income individuals are at greater risk for low diet quality, and our study demonstrated that anxiety may be another important risk factor for healthy eating behaviours in both mothers and toddlers living in low‐income households. Although mothers in our study were not clinically anxious (thus representative of a community sample), mothers who had higher than the average anxiety scores also had lower diet quality for their toddlers and themselves. Our findings are aligned with existing theories (Rosenbaum & White, 2013) and research suggesting that individuals may consume unhealthier foods to relieve anxiety symptoms (Paans et al., 2019; Pallister & Waller, 2008). In turn, individuals on diets low in micronutrients (DiGirolamo et al., 2010) or on high fat diets may display increased anxiety‐like behaviours (Sharma, Fernandes, & Fulton, 2012), illustrating a possible feedback loop among anxiety, food intake, and weight gain (Singh, 2014).

Toddlerhood is a period marked by several developmental changes, as toddlers gain more independence and control especially during mealtimes (Black & Aboud, 2011). Rejection of foods that are novel (food neophobia) is commonly experienced by toddlers, and repeated food exposure is recommended to progress past neophobia (Cole et al., 2017). However, the rejection of foods and irregular eating patterns may be an important source of anxiety for mothers (Farrow & Blissett, 2006), particularly mothers in low‐income households who have financial and food waste concerns. Conversely, maternal anxiety has also been described as a risk factor for picky eating (de Barse et al., 2016) and possibly mediates the relation between child temperament and child eating behaviours (Bergmeier, Skouteris, Horwood, Hooley, & Richardson, 2014). Our study demonstrated that higher maternal anxiety scores were associated with poor toddler diet quality, and this association remained constant over time. Among the few longitudinal studies that have investigated this association, a Norwegian cohort found that mothers with high depression, anxiety, and low self‐esteem were more likely to have a young child (18 months through 3 years of age) eating less wholesome and more unhealthy diets (Ystrom, Niegel, & Vollrath, 2009), an association that was partially mediated by nonresponsive feeding practices, such as restriction or pressure to eat (Ystrom et al., 2012). Public health and primary care initiatives should be designed to help parents understand typical developmental transitions in toddlerhood, especially those associated with mealtime events in order to avoid negative parenting behaviours associated with anxiety and children's eating patterns. Guidelines for anxiety‐reducing strategies during mealtime and general behaviours of toddlerhood may alleviate caregiver's concerns during this transitory phase. Future clinical and research protocols should consider including videotaped feeding and play observations, and anxiety and socioemotional behaviour assessments among both children and caregiver to inform interventions and clinical recommendations.

Our within‐ and between‐person analyses suggested that maternal anxiety may not be an occasion‐dependent risk factor for toddler diet quality but a persistent risk factor; that is, mothers who had on average higher anxiety scores relative to others were more likely to have toddlers who were eating less nutritious foods. However, screening anxiety every 6 months may not have fully captured its variability over time (within‐person variance = 4.6 vs. between‐person variance = 10.7), possibly explaining the null association for the within‐persons effect. It is also possible that between‐ and within‐person levels of anxiety are associated with toddler diets through different mechanisms. Our study provides a first step towards disaggregating within‐ and between‐person sources of variance in the association between maternal anxiety score and toddler diet quality. Future longitudinal studies with repeated measures of anxiety and diet should consider assessing anxiety on a more frequent timescale (i.e., daily, weekly, or monthly) to inform temporal specificity of anxiety that is related to dietary intake. More intensive longitudinal data will not only be able to better estimate within‐ and between‐effects of anxiety and diet, but also be able to inform directionality of the association (e.g., does anxiety predict diet or diet predict anxiety?).

Little attention has been given to health care services and policies among mothers of toddlers (Cheng, Fowles, & Walker, 2006). WIC provides perinatal care for low‐income women, extended to 6 months for nursing mothers, and supplementary food and nutritional counselling for children younger than 5 years. Although the DGA has traditionally provided evidence‐based nutritional information to healthy individuals 2 years and older (Millen et al., 2016), the 2020 edition will aim to cover an important gap by including guidelines for pregnant women as well as infants and toddlers (Altman et al., 2015; Raiten, Raghavan, Porter, Obbagy, & Spahn, 2014). Future revisions will focus not only on the types of foods and nutrients needed during the first 1,000 days (English et al., 2019), but also on caregiver feeding practices that affect the transition to family foods and address both child and caregiver factors that exacerbate picky eating (Spill et al., 2019; Spill et al., 2019). Thus, promotion of anxiety‐reducing strategies for maternal and toddler care and feeding behaviour guidelines may be beneficial for improving maternal and child health.

This study has some limitations. First, our data preclude us from establishing causal associations, and reverse causality might be possible when interpreting our findings (as poor diet may also contribute to higher anxiety). Future longitudinal studies should include more intensive multiple assessment points with shorter “distance” between repeated measures taken on the same subject to explore reverse causality bias. Second, the assessment of diet quality might be susceptible to recall bias. Moreover, diet quality was based on 1 day of intake for mothers and toddlers for each time point. Although use of maternal report of child dietary behaviour for young children is standard, biased responses have been seen among mothers who experience mental health symptoms (Boyle & Pickles, 1997). Third, anxiety was assessed by a short form of the STAI state anxiety scale, which examines generalized anxiety and not anxiety related to parenting. However, the STAI is a widely acceptable general measure, and validity has been established in our study population (Paul, Downs, Schaefer, Beiler, & Weisman, 2013). It is worth noting that the anxiety in this study was relatively mild and not within a clinical range. Fourth, it is possible that our inclusion criteria (no low birthweight or prematurity) may have excluded some women at risk for anxiety and resulted in underrepresentation of those women.

## 5 CONCLUSIONS

In conclusion, our results show that among a sample of mothers with toddlers from low‐income households, mothers with higher anxiety scores have lower diet quality compared with mothers with lower anxiety scores relative to others, especially when toddlers are 12–30 months old. In addition, toddlers of mothers who on average experience higher anxiety scores than others presented lower diet quality, and this relation was consistent across time. Our sample of low‐income mothers and toddlers are not meeting national dietary recommendations and are at risk for developing diet‐related chronic diseases. Future interventions and programmes should aim to improve nutritional quality among this vulnerable population in parallel with anxiety‐reducing approaches that could be beneficial for parenting and feeding during toddlerhood.

## CONFLICTS OF INTEREST

The authors declare that they have no conflicts of interest.

## CONTRIBUTIONS

MMB designed the research; ACBT analysed, interpreted the data, wrote the first draft of the manuscript, and had the primary responsibility for the final content; YW provided statistical guidance in data analyses; MMB and YW assisted in the interpretation of results; and MMB, PJS, and KMH provided expertise on maternal psychological well‐being and feeding practices, thus shaping the content of Sections 1, 2, and 4. All authors participated in the preparation of the paper and critically reviewed all sections of the text for important intellectual content.

## Supporting information


**Table S1.** Total Healthy Eating Index (HEI 2015) and components of HEI for mother and toddler over time.

**Table S2.** Associations between maternal anxiety score and diet quality of mothers and toddlers across three time points.
