# Supplementary material for: Maternal anxiety and diet quality among mothers and toddlers from low‐income households
Source: Matern Child Nutr. 2020 Mar 8;16(4):e12992. doi: 10.1111/mcn.12992 (PMC7507505; doi:10.1111/mcn.12992)
Supplement: Supplementary file 1 — Table S1. Total Healthy Eating Index (HEI 2015) and components of HEI for mother and toddler over time. [file MCN-16-e12992-s001.docx]

**Supplemental Table 1.** Total Healthy Eating Index (HEI 2015) and components of HEI for mother and toddler over time.

| Maternal HEI | Possible Range | Time 1 | Time 2 | Time 3 |
| --- | --- | --- | --- | --- |
|  |  | Mean (SD) | Mean (SD) | Mean (SD) |
| Total vegetables | 0-5 | 2.80 (1.62) | 2.85 (1.61) | 2.84 (1.65) |
| Greens and beans | 0-5 | 1.27 (2.03) | 1.21 (1.99) | 1.08 (1.87) |
| Total fruits | 0-5 | 2.49 (2.09) | 2.44 (2.05) | 2.30 (2.08) |
| Total whole fruits | 0-5 | 1.71 (2.11) | 1.85 (2.17) | 1.57 (2.09) |
| Whole grains | 0-10 | 1.94 (2.82) | 2.28 (3.03) | 2.29 (3.26) |
| Total dairy | 0-10 | 4.39 (3.23) | 4.83 (3.23) | 4.74 (3.34) |
| Total protein foods | 0-5 | 4.08 (1.36) | 4.11 (1.34) | 4.16 (1.32) |
| Seafood and plant proteins | 0-5 | 1.52 (2.03) | 1.83 (2.17) | 1.78 (2.18) |
| Fatty acids | 0-10 | 4.80 (3.38) | 5.11 (3.43) | 4.99 (3.50) |
| Sodium | 0-10 | 5.26 (3.70) | 4.20 (3.35) | 4.11 (3.53) |
| Refined grains | 0-10 | 6.41 (3.61) | 6.01 (3.73) | 6.06 (3.68) |
| Saturated fats | 0-10 | 5.90 (3.29) | 5.99 (3.16) | 5.96 (3.23) |
| Added sugars | 0-10 | 5.42 (3.68) | 5.58 (3.60) | 5.64 (3.48) |
| HEI 2015 total score for mothers | 0-100 | 47.99 (12.10) | 48.30 (12.11) | 47.51 (13.24) |
| Toddler HEI |  |  |  |  |
| Total vegetables | 0-5 | 1.94 (1.47) | 2.28 (1.63) | 2.09 (1.48) |
| Greens and beans | 0-5 | 0.69 (1.51) | 0.82 (1.64) | 0.82 (1.70) |
| Total fruits | 0-5 | 3.95 (1.61) | 3.98 (1.69) | 3.85 (1.73) |
| Total whole fruits | 0-5 | 3.13 (2.16) | 3.06 (2.25) | 2.65 (2.28) |
| Whole grains | 0-10 | 2.81 (3.41) | 2.80 (3.20) | 3.12 (3.61) |
| Total dairy | 0-10 | 7.74 (3.27) | 7.20 (3.32) | 6.53 (3.51) |
| Total protein foods | 0-5 | 3.41 (1.52) | 3.54 (1.59) | 3.64 (1.47) |
| Seafood and plant proteins | 0-5 | 1.30 (1.91) | 1.46 (1.94) | 1.76 (2.13) |
| Fatty acids | 0-10 | 2.75 (3.26) | 3.57 (3.22) | 4.03 (3.29) |
| Sodium | 0-10 | 6.81 (3.29) | 5.43 (3.52) | 5.33 (3.42) |
| Refined grains | 0-10 | 7.44 (3.27) | 6.86 (3.39) | 6.33 (3.55) |
| Saturated fats | 0-10 | 4.40 (3.56) | 5.36 (3.29) | 6.14 (3.16) |
| Added sugars | 0-10 | 7.69 (2.79) | 7.02 (3.17) | 6.33 (3.30) |
| HEI 2015 total score for toddlers | 0-100 | 54.07 (10.58) | 53.38 (11.55) | 52.62 (11.79) |

Abbreviations: HEI (healthy eating index); SD (standard deviation)
