# Supplementary material for: Maternal anxiety and diet quality among mothers and toddlers from low‐income households
Source: Matern Child Nutr. 2020 Mar 8;16(4):e12992. doi: 10.1111/mcn.12992 (PMC7507505; doi:10.1111/mcn.12992)
Supplement: Supplementary file 2 — Table S2. Associations between maternal anxiety score and diet quality of mothers and toddlers across three time points. [file MCN-16-e12992-s002.docx]

**Supplemental Table 2**. Associations between maternal anxiety score and diet quality of mothers and toddlers across three time points.

| Model | Maternal Anxiety Score | | | | | | | |
| --- | --- | --- | --- | --- | --- | --- | --- | --- |
|  | Anxiety | | Difference in beta between time 1 and time 2 | | Difference in beta between time 1 and time 3 | | Difference in beta between time 2 and time 3 | |
| **Maternal HEI-2015 total score**^a^ | b | 95% CI | b | 95% CI | b | 95% CI | b | 95% CI |
| Time 1 | -0.71 | -1.09, -0.34 | reference | | reference | |  |  |
| Time 2 | -0.51 | -0.97, -0.05 | 0.20 | -0.35, 0.76 |  |  | reference | |
| Time 3 | -0.143 | -0.54, 0.27 |  |  | 0.58 | 0.05, 1.11 | 0.37 | -0.20, 0.96 |
| **Toddler HEI-2015 total score**^a^ |  |  |  |  |  |  |  |  |
| Time 1 | -0.51 | -0.87, -0.15 | reference | | reference | |  |  |
| Time 2 | 0.01 | -0.44, 0.45 | 0.52 | -0.04, 1.09 |  |  | reference | |
| Time 3 | -0.17 | -0.57, 0.22 |  |  | 0.34 | -0.18, 0.87 | -0.17 | -0.76, 0.41 |

Abbreviations: HEI (Healthy Eating Index 2015); SD (standard deviation); CI (confidence interval)

b represents the average marginal effect of change in HEI-2015 total score that is produced by a 1-unit increase in maternal anxiety at a given time point.

Time 1 (enrollment), Time 2 (6-month follow-up), Time 3 (12-mo follow-up)

^a^ Hierarchical models controlled for maternal and toddler’s age and sex, maternal body mass index (kg/m^2^), toddler BMI z-score, living at or below the poverty line, parity, age of youngest child, and intervention group.
